# Technical assessment of different extraction methods and transcriptome profiling of RNA isolated from small volumes of blood

**DOI:** 10.1038/s41598-023-30629-5

**Published:** 2023-03-03

**Authors:** Mahesh Kumar Reddy Kalikiri, Harshitha Shobha Manjunath, Fazulur Rehaman Vempalli, Lisa Sara Mathew, Li Liu, Li Wang, Guishuang Wang, Kun Wang, Oleksandr Soloviov, Stephan Lorenz, Sara Tomei

**Affiliations:** 1 Clinical Genomics Laboratory, Integrated Genomics Services, Sidra Medicine, Doha, Qatar; 2 Omics Core, Integrated Genomic Services, Sidra Medicine, Doha, Qatar; 3 Bioinformatics Core, Integrated Genomics Services, Sidra Medicine, Doha, Qatar

**Keywords:** Biological techniques, Biotechnology, Computational biology and bioinformatics, Molecular biology

## Abstract

Transcriptome profiling of human whole blood is used to discover biomarkers of diseases and to assess phenotypic traits. Recently, finger-stick blood collection systems have allowed a less invasive and quicker collection of peripheral blood. Such non-invasive sampling of small volumes of blood offers practical advantages. The quality of gene expression data is strictly dependent on the steps used for the sample collection, extraction, preparation and sequencing. Here we have: (i) compared the manual and automated RNA extraction of small volumes of blood using the Tempus Spin RNA isolation kit and the MagMAX for Stabilized Blood RNA Isolation kit , respectively; and (ii) assessed the effect of TURBO DNA Free treatment on the transcriptomic data of RNA isolated from small volumes of blood. We have used the QuantSeq 3′ FWD mRNA-Seq Library Prep kit to prepare RNA-seq libraries, which were sequenced on the Illumina NextSeq 500 system. The samples isolated manually displayed a higher variability in the transcriptomic data as compared to the other samples. The TURBO DNA Free treatment affected the RNA samples negatively, decreasing the RNA yield and reducing the quality and reproducibility of the transcriptomic data. We conclude that automated extraction systems should be preferred over manual extraction systems for data consistency, and that the TURBO DNA Free treatment should be avoided when working on RNA samples isolated manually from small volumes of blood.

## Introduction

Transcriptome profiling is a reference research field, and it is applied especially for the study of human diseases^1,2^. The analysis of the human transcriptome allows us to understand the human genome at the gene expression level and also provides a window to understand gene regulation and genome plasticity^2–4^. However, gene expression profiling can only be of value when the RNA under study is representative of the starting material^5^. Unfortunately, several pre-analytical factors affect the RNA yield and quality and might hamper the representativeness of the starting RNA^5^, including RNA isolation methods, DNase treatments, library preparation etc. The ex vivo instability of RNA can be reduced if the blood is freshly extracted and processed for RNA isolation immediately. However, this is not a feasible option and, in most cases, blood is collected with variations in timing and storage conditions, which have been proven to affect transcriptomic profiles to some degree^6^. Different RNA stabilizers are employed to overcome the limitation of using fresh blood for RNA isolation^7–9^. Such stabilizer solutions immediately lyse cells chemically and stabilize nucleic acids. Cellular RNases are inactivated, and the RNA is selectively precipitated, leaving proteins and genomic DNA in solution. One of the most common RNA stabilizer solutions is represented by Tempus Blood RNA (Thermo Fisher Scientific, MA, USA). Tempus system uses a solid-phase, silica-based isolation strategy and its performance has been proven higher than other systems^10^. Yet, Tempus Blood RNA utility is limited by the requirement of a venous blood samples of at least 3.0 ml. Recently, finger-stick blood collection systems have made it possible to collect peripheral blood without the need of medical infrastructures, offering practical and logistic advantages^11^. Nevertheless, technical improvements are required to make the gene expression profiling of small volumes of blood a reliable and reproducible technique^12^. Automated workflows offer several advantages for large-scale projects, as they increase sample throughput and reduce cost and manual errors^13–16^. The MagMAX for Stabilized Blood RNA Isolation kit (Thermo Fisher Scientific, MA, USA) employs a magnetic bead-based technology to purify RNA from blood stored in Tempus solution. Because of its bead-based approach, it can easily be implemented on automated systems. The MagMax workflow includes a TURBO DNase step that removes contaminating DNA and can also be implemented in automation systems, such as the KingFisher Magnetic Particle Processors (Thermo Fisher Scientific, MA, USA). However, there currently exist many liquid-handling workstations on the market, each one of them offers different degrees of flexibility. Hamilton Robotics (Hamilton, NV, USA), for instance, offers autonomous programming^15^. In this study the Hamilton NGS Star platform has been employed for automated RNA extraction.

Here, we have compared the manual RNA isolation of small volumes of blood (Tempus Blood RNA kit) and an automated workflow implemented in-house by using the MagMAX for Stabilized Blood RNA Isolation kit on the Hamilton NGS Star platform (Hamilton, NV, USA); we have also evaluated the effect of the TURBO DNA Free treatment (Thermo Fisher Scientific, MA, USA) on the reproducibility and reliability of the transcriptomic data. Transcriptome sequencing was performed by using the Lexogen QuantSeq 3′ mRNA-Seq Library Prep FWD kit (Lexogen GmbH, Austria) with unique molecular identifiers (UMI), because of its streamlined protocol and its relatively lower cost as compared to other systems.

Here we demonstrate that the automated extraction workflow produces more consistent data as compared to the manual extraction method and that the TURBO DNA Free treatment should be avoided when working on RNA isolated manually from small volumes of blood.

## Methods

### RNA isolation

**Figure 1:**
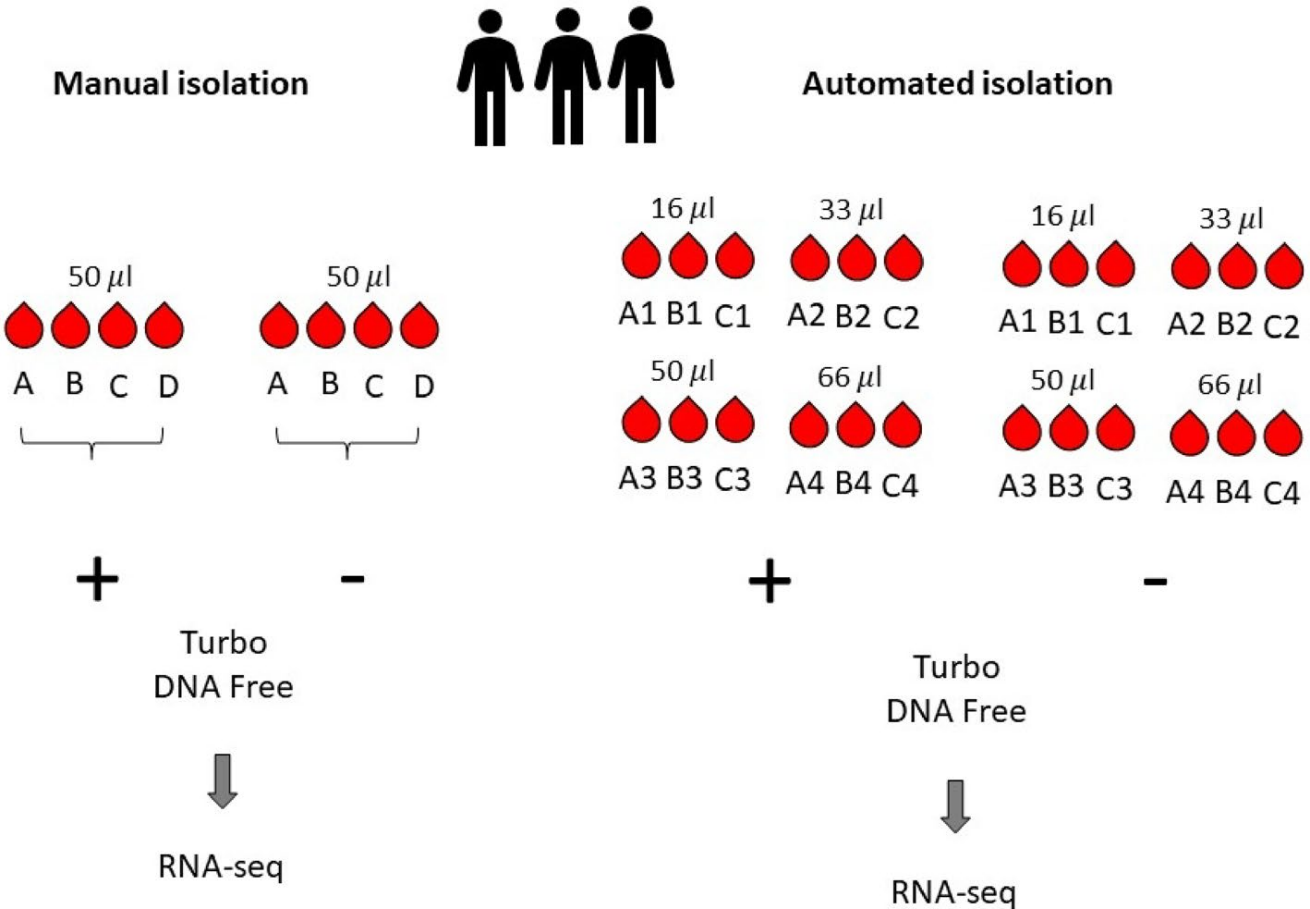
Outline of the RNA samples isolated in this study for the manual and automated workflows.

**Figure 2:**
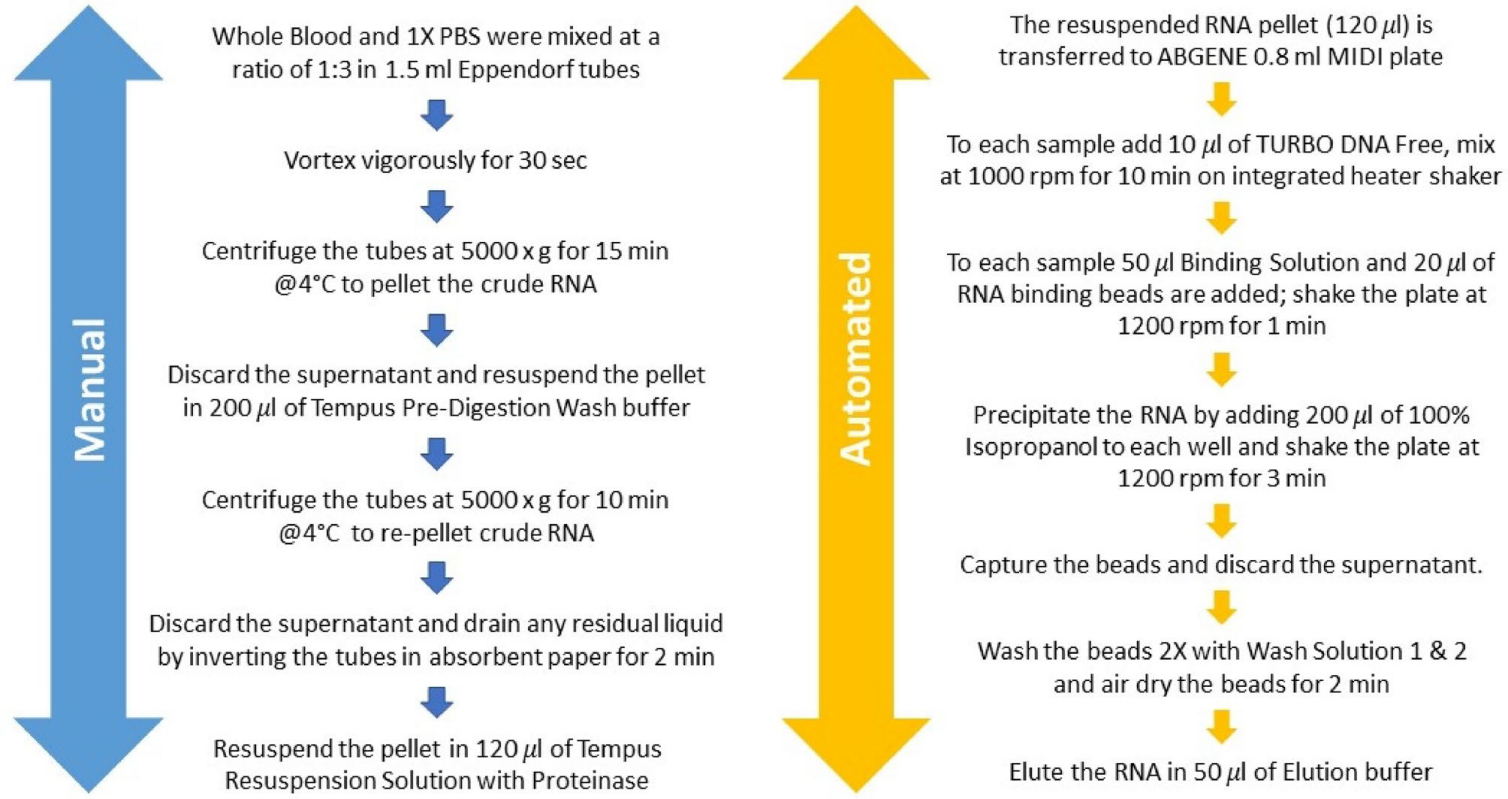
Overview of the manual (blue) and automated (yellow) steps included in the in-house RNA extraction of the MagMax workflow.

Whole blood was collected from healthy donors as previously described^11^. Ethical approvals were collected from Sidra Institutional Review Board committee (IRB Protocol #1707011887). An informed consent was obtained from the study subjects and all methods were performed in accordance with the relevant guidelines and regulations. Different conditions were tested for each healthy donor recruited, as shown in Fig. 1. For the manual process, the Tempus Spin RNA Isolation kit was used to isolate and purify RNA from blood collected in the capillary tubes according to the manufacturer’s instructions and adjusting the reagents volumes to maintain the working ratios required by the protocol. For the automated process, the MagMAX for Stabilized Blood RNA Isolation kit was used on the Hamilton NGS Star platform using a protocol developed in-house. The protocol developed in house includes some initial manual steps. Figure 2 summarizes the manual and automated steps of the protocol developed in-house with the MagMAX for Stabilized Blood RNA Isolation kit . Supplementary Fig. 1 displays the deck layout of the Hamilton NGS STAR. The MagMAX for Stabilized Blood RNA Isolation kit uses a magnetic bead-based technology and includes a DNase treatment step (TURBO DNA Free treatment). After extraction, RNA was quantified on the NanoDrop 8000 Spectrophotometer (Thermo Fisher Scientific, MA, USA) to evaluate the concentration and purity. The amount of RNA present in each sample was then detected on the Qubit 2.0 Fluorometer (Thermo Fisher Scientific, MA, USA) using the Qubit RNA HS Assay kit (Thermo Fisher Scientific, MA, USA). The RNA profile and integrity of all samples was assessed using the RNA Assay Reagent kit on the LabChip GXII (PerkinElmer, MA, USA). Samples were evaluated according to their RIN (RNA integrity number). This score is classified on a numbering system from 1 to 10, with 1 indicating the most degraded RNA and 10 indicating the most intact RNA.

### Lexogen QuantSeq 3′ mRNA-Seq

Lexogen QuantSeq3′ mRNA-Seq libraries for Illumina sequencing were prepared from 120 ng of total RNA according to the manufacturer’s recommendations.

The first strand was synthesized by reverse transcription with oligo-dT priming followed by treatment with The Globin Block (RS-GB) Module for QuantSeq. The RS-GB solution has specific oligos which selectively bind to the globin mRNA cDNA-transcripts and prevent the generation of library fragments from globin mRNAs, by blocking their extension during second strand synthesis, initiated by random priming. Because the globin blocking oligo is bound close to the poly-(T)-section of the first strand, the second strand synthesis stops for globin transcripts and does not reach the 5′ sequencing tag of the first strand, thus yielding non-amplifiable globin cDNAs. The non-globin tagged double-stranded cDNA library fragments were then amplified for 18 PCR cycles and labelled with different single indices. The UMI Second Strand Synthesis Mix (USS) containing Unique Molecular Identifiers (UMIs) was used during second strand synthesis prior to PCR. UMIs act as tags that allow detection and removal of PCR duplicates in sequencing data.

The quality and size of the libraries were determined using the NGS 3K assay on the Labchip GXII and pooled based on quantification via qPCR using the KAPA HiFi Library quantification kit on the LightCycler 480 II (Roche Diagnostics, Basel, Switzerland). Libraries were pooled on the Hamilton MicroLab Star and sequenced on the Illumina NextSeq 500 system at a depth of 8 million reads per library. Out of the 60 samples, 48 samples were sequenced using the High Output 75 cycles kit and single end read mode and 12 samples were sequenced using the High Output 150 cycles kit and single end read mode (Supplementary Table 1). The QC of sequencing data was performed as recommended by Illumina.

### Data analysis

The preliminary quality of sequencing reads was assessed using FASTQC (v.0.11.8). We have used the recommended QuantSeq FWD-UMI Data Analysis Pipeline which is specific for Quantseq FWD libraries that contain UMIs. First, the umi2index process adds the 6 nucleotide UMI sequence to the identifier of each read and trims the UMI from the start of each read; the FASTQ file generated at this step was processed through trimming and alignment. Quality trimming was performed to remove the adapter sequences and polyA tails using bbduk.sh from BBMap (v38.69). Per base sequence quality plots showing average quality scores above 30 for raw fastq and the quality trimmed fastq is reported in Supplementary Fig. 3. Trimmed reads were mapped to the human genome GRCh38.p13 (Genome Reference Consortium Human Build 38, INSDC Assembly GCA_000001405.28, Dec 2013) using STAR_2.6.1d aligner and HTSeq-count (v0.9.1) was used to generate the raw counts. The reads of the samples sequenced at 150 nt were trimmed to the first 75 nt. As per Lexogen recommendations, sequencing at 150 nt and trimming to the first 75 nt gives the same results as sequencing at 75 nt. The RSeQC distribution was similar for the untrimmed and trimmed reads (Supplementary Fig. 2).

Normalized data was transformed using variance-stabilizing transform (VST) and removed batch effect using limma::removeBatchEffect from Lima package (v3.48.2). The RSeQC (v3.0.1) geneBody_coverage2.py and the RSeQC’s read_distribution.py modules were used to calculate the RNA-seq reads coverage over the gene body and the distribution of mapped reads across genomic features, respectively. Heatmaps, correlation matrices and PCA plots were generated as relevant by using R packages.

A simulation analysis was performed using RSEM (v1.2.25). Reads of 75 nt were simulated to different depths, namely: 50 M, 100 M, and 400 M reads using rsem-simulate-reads. This program took estimated_model_file and estimated_isoform_results as input which were learned from real data using rsem-calculate-expression program. Simulated reads were analyzed in the same way as for the real data (aligned and quantified using STAR (v2.1.6_d) and HTSeq-count (0.9.1) tools followed by DeSeq2 VST normalization and generate QC plots).

Non-parametric Wilcoxon signed-ranks and Mann–Whitney tests were applied to compare groups as appropriate. Non-parametric Spearman *r* test was used to evaluate correlations. All statistical tests were two-sided. p-values lower than 0.05 were considered statistically significant.

## Results

### RNA quality and quantity

**Figure 3:**
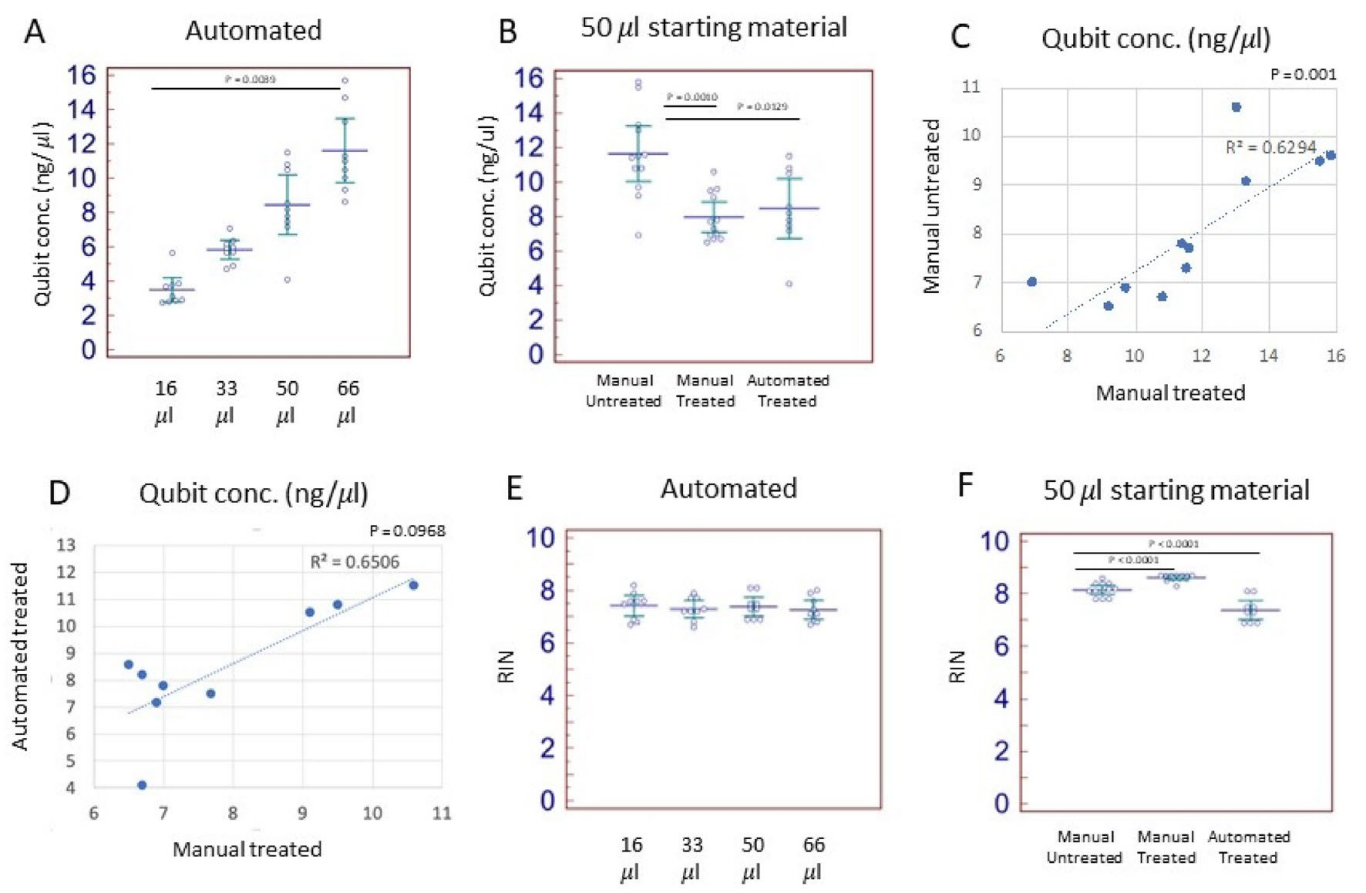
RNA concentration (Qubit, ng/μl) of the automated workflow according to sample volume (**A**). RNA concentration (Qubit, ng/μl) of the 50 μl blood samples according to the extraction method (**B**). Correlation plot of the concentration values of the samples isolated manually as DNA Free-treated and untreated (**C**). Correlation plot of the concentration values of the samples DNA Free-treated isolated manually and on the automation system (**D**). RIN values of the samples processed on the automated workflow according to their volume (**E**). RIN values of the samples processed from 50 μl of blood for the different isolation methods (**F**).

Samples were divided into 6 groups, according to their (i) extraction method (manual vs automated); (ii) TURBO DNA Free treatment (treated vs untreated), and iii. starting volume of whole blood (16 μl, 33 μl, 50 μl and 66 μl). The elution volume was 50 μl for all the extractions performed. Thus, we have used the Qubit (fluorescence-based) concentration values (ng/μl) for comparative analysis. As expected, the RNA concentration increased parallelly to the increased volume of blood used for the extraction, with the concentration obtained from 66 μl of blood being significantly higher as compared to the concentration obtained from 16 μl of blood (Wilcoxon matched-pairs signed-ranks test, p = 0.0039, Fig. 3A, Table 1). Interestingly, the variability (measured by the standard deviation) increased parallelly to the amount of whole blood used for the extraction (Fig. 3A, Table 1), suggesting that the sampling might give more consistent results for lower volumes of blood.

**Table 1:** Summary of the RNA QC metrics according to the sample groups.

Group	Qubit Concentration (ng/μl; Average ± SD)	RIN (average ± SD)	A260/A230 (average ± SD)	A260/A280 (average ± SD)
Manual untreated 50 μl blood	11.62 ± 2.53	8.16 ± 0.26	1.45 ± 0.55	2.14 ± 0.14
Manual TURBO treated 50 μl blood	7.95 ± 1.39	8.62 ± 0.12	1.99 ± 1.45	1.31 ± 0.09
Automated TURBO treated 16 μl blood	3.50 ± 0.92	7.42 ± 0.51	0.63 ± 0.29	2.19 ± 0.41
Automated TURBO treated 33 μl blood	7.82 ± 0.72	7.29 ± 0.42	1.04 ± 0.38	2.24 ± 0.21
Automated TURBO treated 50 μl blood	8.45 ± 2.27	7.39 ± 0.47	1.2 ± 0.17	2.06 ± 0.23
Automated TURBO treated 66 μl blood	11.61 ± 2.44	7.26 ± 0.47	1.16 ± 0.29	2.12 ± 0.09

When using 50 μl of blood, we found a significant decrease of the RNA concentration in the manual TURBO-treated protocol and the automated TURBO-treated protocol as compared to the manual untreated protocol (Wilcoxon matched-pairs signed-ranks test, p = 0.0010 and p = 0.0129, respectively, Fig. 3B). While the RNA concentration values correlated significantly for the manual treated and untreated protocols (Spearman *r* test, p = 0.001) we found no significant correlation between the manual and automated protocols that included the TURBO DNA Free treatment, suggesting that workflow-specific steps might affect the RNA concentration more than the TURBO DNA Free treatment and biological variables (i.e. individual cell counts, Fig. 3C,D).

Overall, all the RNA isolated was of good quality. No significant difference in RIN value was observed across samples processed from different volumes of starting material (Fig. 3E). Nevertheless, the RIN values obtained from the different samples varied across the experimental groups with the manual extraction method producing overall higher RIN values as compared to the automated methods (Fig. 3F, Table 1).

The A260/A230 values varied across the experimental groups with the automated TURBO-treated samples of 16 μl blood producing the lowest A260/A230 ratio (0.63 ± 0.29). The A260/A280 values were > 2 for all the experimental groups except for the manual TURBO-treated 50 μl blood samples that displayed an average A260/A280 ratio of 1.31 ± 0.09.

We next sought to assess the effect of TURBO DNA Free treatment on the RNA yield and RIN values. For the manual protocol the TURBO DNA Free treatment resulted in an overall yield reduction > 25% (Table 2), while the RIN values increased slightly (Table 2). When we compared the yield and RIN values in the automated TURBO DNA Free protocol to the manual untreated protocol, we found a yield reduction similar to the one induced by the TURBO DNA Free treatment in the manual protocol (Table 3). However, the TURBO DNA Free treatment induced a RIN reduction between 3.36–11.51% in the automated protocol as compared to the manual untreated protocol (Table 3).

**Table 2:** RNA yield reduction and RIN increase induced by TURBO DNA Free treatment in the manual protocol.

Subject ID	Average yield (ng) manual untreated	Average yield (ng) manual treated	Yield reduction (%)	Average RIN manual untreated	Average RIN manual treated	RIN increase (%)
S1	495.00	367.50	25.76	8.33	8.70	4.50
S2	720.00	485.00	32.64	7.98	8.50	6.58
S3	528.75	340.00	35.70	8.18	8.68	6.12

**Table 3:** RNA yield and RIN reduction induced by TURBO DNA Free treatment in the automated protocol (50 μl blood).

Subject ID	Average yield (ng) manual untreated	Average yield (ng) automated treated	Yield reduction (%)	Average RIN manual untreated	Average RIN automated treated	RIN reduction (%)
S1	495.00	373.83	24.48	8.33	7.37	11.51
S2	720.00	546.67	24.07	7.98	6.90	13.48
S3	528.75	347.17	34.34	8.18	7.90	3.36

### Gene expression analyses

We assessed the reproducibility of gene expression profiles obtained from the different RNA extraction methods. Out of the 60 samples, 2 samples extracted manually from the same donor generated a library of a size and concentration deviating from what is recommended for Lexogen Quant-Seq 3′mRNA-Seq Library Prep and were removed from the downstream processing. This could be due to the low purity of the samples as their Nanodrop readings demonstrated a high A260/A230 ratio. Two additional samples produced libraries of suboptimal molarity and were labeled as “low conc. library” for further analyses. After sequencing and read mapping, we have evaluated the alignment scores, the count assignments and read distribution across the samples. Because in the Lexogen Quant-Seq 3′mRNA-Seq Library Prep method cDNA molecules are transcribed from the 3′ end of the mRNAs, the reads preferentially mapped to the 3′ end of the transcript. Samples processed manually had a slightly higher percentage of reads mapping to exonic region, however the samples showed overall consistent body coverage, alignment scores and gene type assignment profiles (Supplementary Fig. 4).

**Figure 4:**
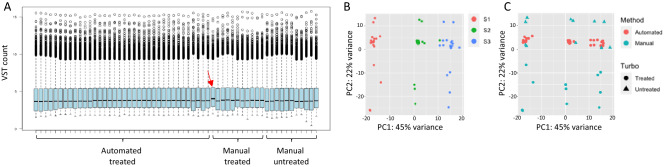
Box plot of the VST (variance stabilizing transformation) count of the sample set. The sample indicated by the red arrow gave a low library yield and displayed a higher VST median as compared to the other samples (**A**). Principal component analysis of the complete datasets; the different individuals are color-coded (**B**). Principal component analysis of the complete datasets; the different extraction methods are color-coded; samples processed with TURBO DNA Free treatment are indicated by the round shape, the untreated samples are indicated by the triangle shape (**C**).

When we looked at the distribution of the VST counts across the sample set, we noticed an overall homogeneous distribution of the VST counts, however the samples processed manually showed a higher variability of the VST counts as compared to the samples processed with the automated method (Fig. 4A). The simulation analysis yielded similar read distribution over gene body elements and gene types across the methods, although the read distribution was more consistent for the samples processed with the automated method (Supplementary Figs. 5 and 6).

To explore the effect of the different variables assessed in the study on the complete transcriptomic data we have used principal component analysis (PCA).

The assignment of the samples to the three individuals accurately predicted their distribution in a three-dimensional space suggesting that their transcriptional signatures can be retraced to the individual biology (Fig. 4B). Contrarily, the different extraction methods and the DNase treatment seemed to have a negligible effect on the sample distribution (Fig. 4C), although samples processed manually displayed a higher variability. This might be explained by the fact that biological variables might have a larger effect on the transcriptomic data as compared to analytical variables (i.e., isolation method, DNase treatment). Similar findings were obtained with the simulation analysis (Supplementary Fig. 7). It should be noted that in the PCA plots displayed in Fig. 4B,C, the variance of PC1 was 45%, indicating that the transcriptomic data of the samples was overall quite similar.

**Figure 5:**
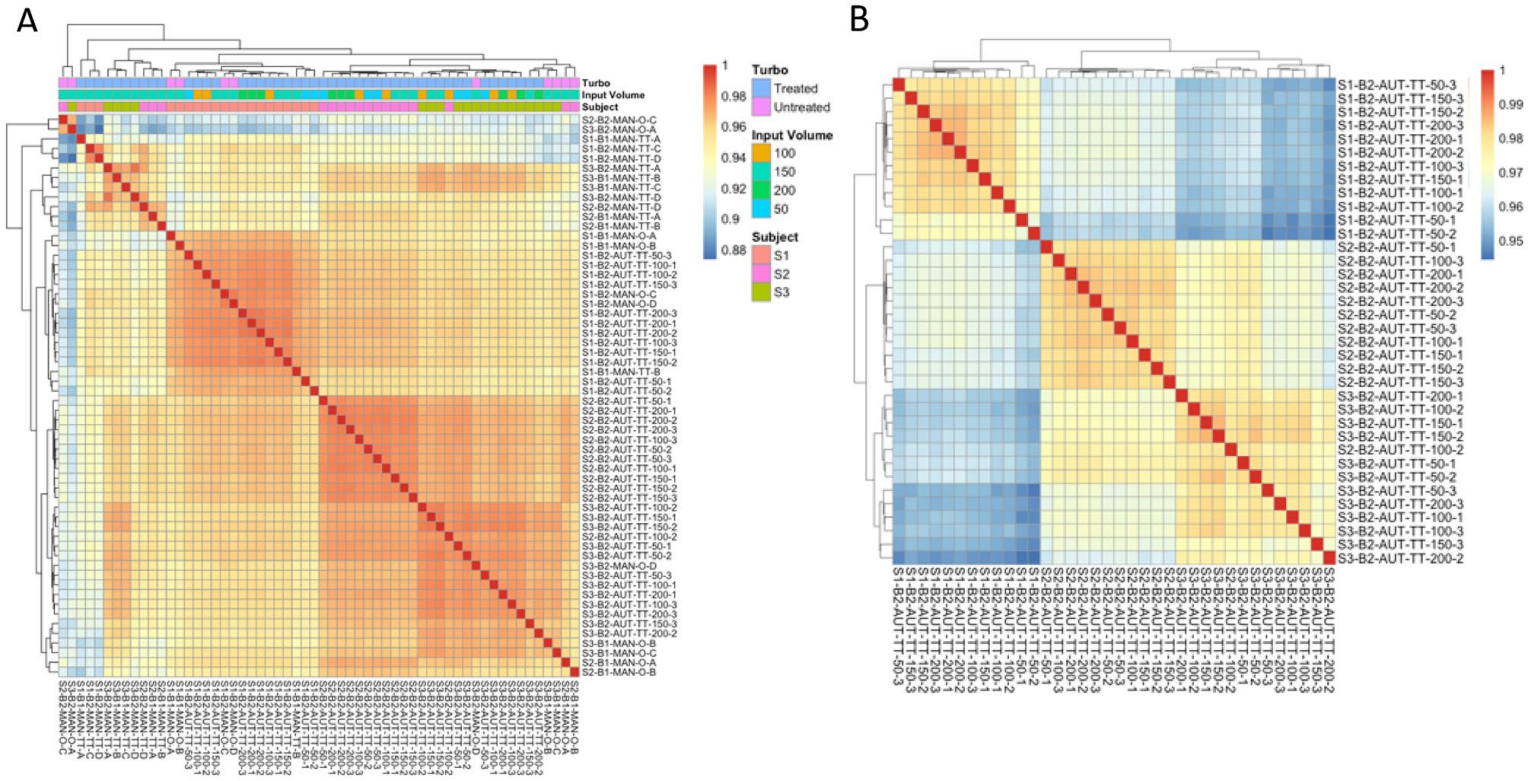
Correlation matrix of the complete sample set (**A**). Correlation matrix of the samples processed with the automation protocol only (**B**). The correlation scale spans from 0.88 (blue color) to 1 (orange color). Overall, the manual and automated samples clustered separately; the assignment of the samples to the individual subjects seemed to drive the correlation. *S*: sample, *B*: batch, *AUT*: automated, *MAN*: manual, *O*: original, *TT*: TURBO Treated. The aliquots of the samples processed manually were labeled as A, B, C, D. The volumes (in μl) used for the samples processed on the automated protocol are reported after the “TT” (50, 100, 150, 200); the numbers reported after the volumes for the samples processed on the automated protocol indicate the different aliquots (1, 2, 3).

Nevertheless, the correlation matrix identified an overall high degree of similarity across the samples isolated with the automation method as compared to the ones isolated manually, irrespective to starting blood volume and DNase treatment (Fig. 5A). When performing correlation analysis only on the samples isolated on the automated system, we found an almost perfect correlation of samples belonging to the same individual, irrespective to the starting blood volume (Fig. 5B), supporting the sampling of volumes of blood as low as 16 μl as an efficient method for whole blood transcriptomic profiling.

### Processing time/sample throughput comparison between the manual and automated workflow

We have also evaluated the processing time of the standard manual extraction protocol and the automated protocol developed in-house on the Hamilton NGS Star platform.

The manual workflow overall takes about 75 min hands-on-time and 65 min incubation time, while the automated workflow overall takes 20 min hands-on-time and 50 min incubation time. The above calculations refer to the processing of a batch of 24 samples. However, the sample throughput can be significantly increased in the automated workflow as the Hamilton NGS STAR system is equipped with 3 × 32 sample tube carriers and it can process 96 samples per batch. Additionally, faster bead clean-up steps can be adopted to this method if the liquid handler is equipped with 96-Multi Probe Head.

## Discussion

Transcriptomic profiling of peripheral blood is often employed for the identification of susceptibility genes or biomarkers of human phenotypes and diseases^7,17,18^. Blood gene expression profiles can be significantly affected by blood collection and RNA isolation methods^10,19–21^. This is mainly due to the differences in the composition of RNA-stabilizing solutions or differences in the chemistries employed by the different RNA isolation methods. As the manual protocol employs spin columns while the automated protocol uses a magnetic beads approach, we questioned whether the use of the two different methods in this study could have impacted the RNA QC and gene expression profiles. Although we found significant differences in RNA quality and yield, overall the gene expression profiles were maintained, and the inter-individual differences were reproducible across the different extraction methods. The transcriptomic profiles were in fact driven mainly by the subject assignment rather than by analytical variables, suggesting that the Lexogen QuantSeq 3′ FWD mRNA-Seq is a robust method for gene expression profiling. The Lexogen QuantSeq 3′ FWD mRNA-Seq has a streamlined protocol, does not require RNA fragmentation before reverse transcription and only detects the 3′ end of the mRNA, thus it has been employed for low input and highly degraded RNA^22–24^. The present study supports the Lexogen QuantSeq 3′ FWD mRNA-Seq application for RNA isolated from small volumes of blood.

Especially when working on small volumes of samples, pipetting accuracy and reproducibility are of critical importance; automated RNA isolation systems reduce manual errors and should ensure a higher data reproducibility^13,25^. Automated solutions are currently applied in many fields of life sciences; especially in genomics, laboratory specialists are streamlining their protocols by using automated workstations^26–28^. Automated solutions help cutting costs associated to manual labor and also help wet-lab specialists who would not have to spend long time processing samples^15^. In our study we questioned whether consistent expression profiles could be obtained from the automated isolation of volumes of blood < 50 μl. We found that volumes of blood samples as low as 16 μl provide reliable transcriptomic profiles; interestingly, these samples displayed the lowest variability, suggesting that our in-house approach could be applied in studies where blood is limited.

Other groups have employed TURBO DNA Free treatment for transcriptomic profiles^29,30^. However, to the best of our knowledge this is the first assessment on the effect of TURBO DNA Free treatment on RNA isolated from small volumes of blood by using a manual and an automated workflow. The TURBO DNA Free treatment impacted more negatively the samples processed manually. The DNase inactivation reagent is in fact known to sequester divalent cations, change the buffer condition and interfere with enzymatic reactions. In our study, when we compared the automated and manual workflows both including the TURBO DNA Free treatment, we found the treatment to have a stronger negative impact on the manual samples, likely because the treatment is performed at the end of the workflow, differently to the automated protocol.

We expect this study to increase the adoption of automation systems for RNA isolation from small volumes of blood especially in core facility settings where sample throughput and turn-around-time are of critical importance.

## Conclusion

Collectively, these results indicate that transcriptomic profiles obtained using the Lexogen QuantSeq 3′ FWD mRNA-Seq protocol are highly reproducible across different extraction methods employed for small volumes of blood, despite differences in RNA quantity and quality. The TURBO DNase treatment should be avoided when isolating RNA from small volumes of blood. The data produced from the automated method displayed less variability as compared to the manual method.

## Supplementary Information

Supplementary Information.

## Data Availability

The datasets generated and analyzed during the current study are available in the Gene Expression Omnibus (GEO) repository, [https://www.ncbi.nlm.nih.gov/geo/query/acc.cgi?acc=GSE210812].
