# Supplementary material for: Technical assessment of different extraction methods and transcriptome profiling of RNA isolated from small volumes of blood
Source: Sci Rep. 2023 Mar 3;13:3598. doi: 10.1038/s41598-023-30629-5 (PMC9984369; doi:10.1038/s41598-023-30629-5)
Supplement: Supplementary file 1 — Supplementary Information. [file 41598_2023_30629_MOESM1_ESM.docx]

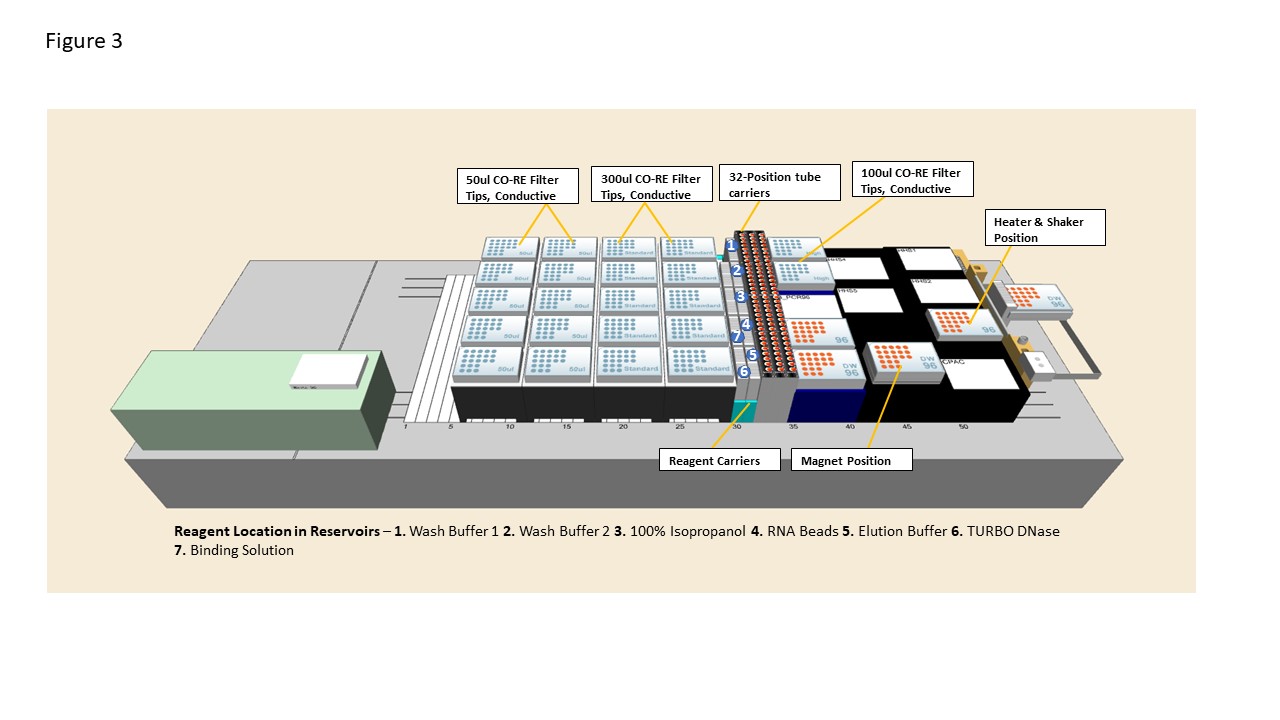


**Supplementary Figure 1.** Deck layout of the Hamilton NGS STAR for the in-house RNA extraction of the MagMax workflow.

**Supplementary Figure 2.** RSeQC Read distribution across genomic elements (3’UTR, 5’ UTR, CDS exons, TSS, Intronic & other intergenic elements) of the 12 samples sequenced at 150 bp read length before and after trimming. “Trim”: trimmed.

**Supplementary Figure 3.** Per base sequence quality plots of the Raw QC (top panel) and Post QC (bottom panel) data.


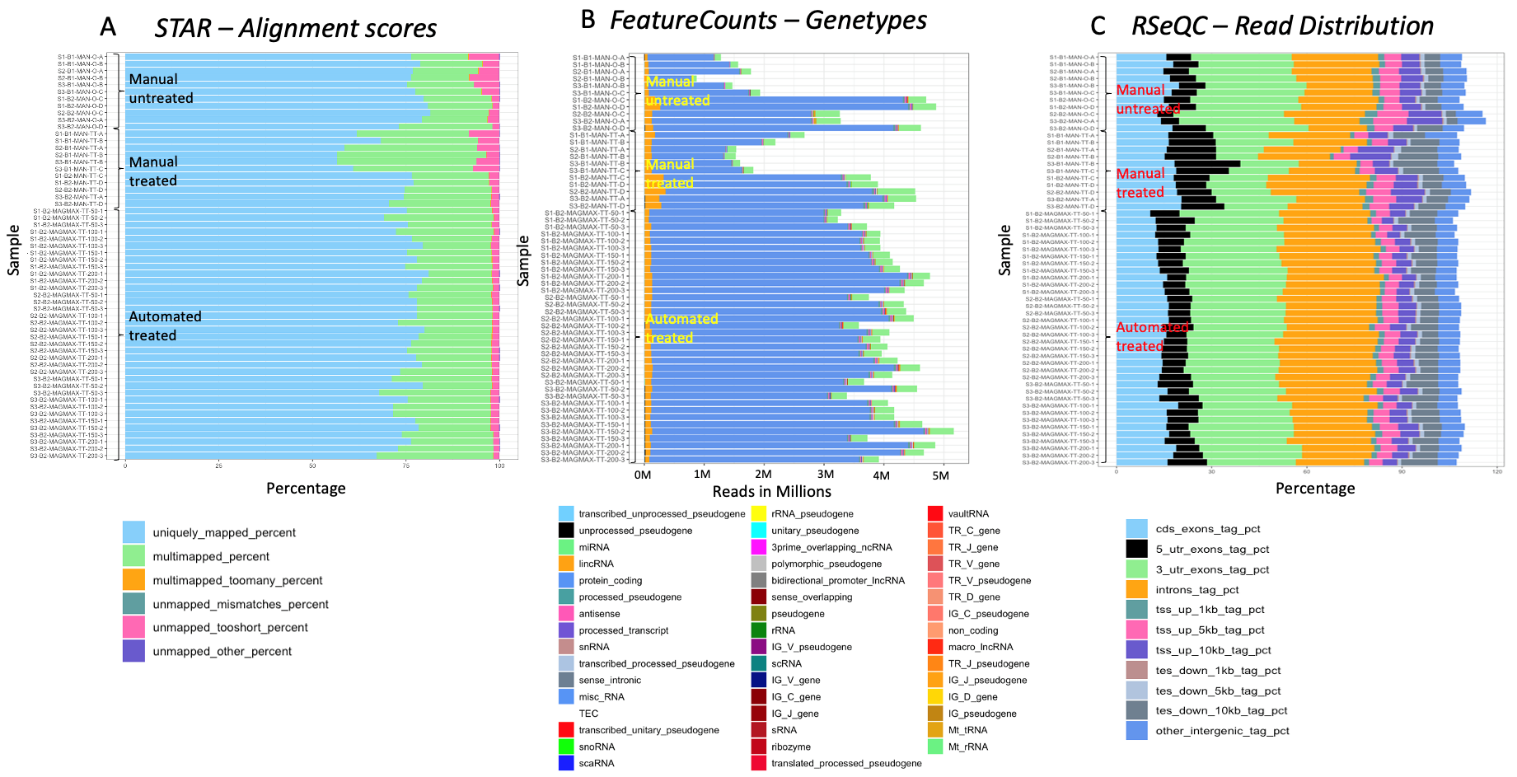


**Supplementary Figure 4.** STAR alignment scores (A), Feature Counts Gene types read distribution (B), and RSeQC read distribution (C) of the sample set according to the extraction method.


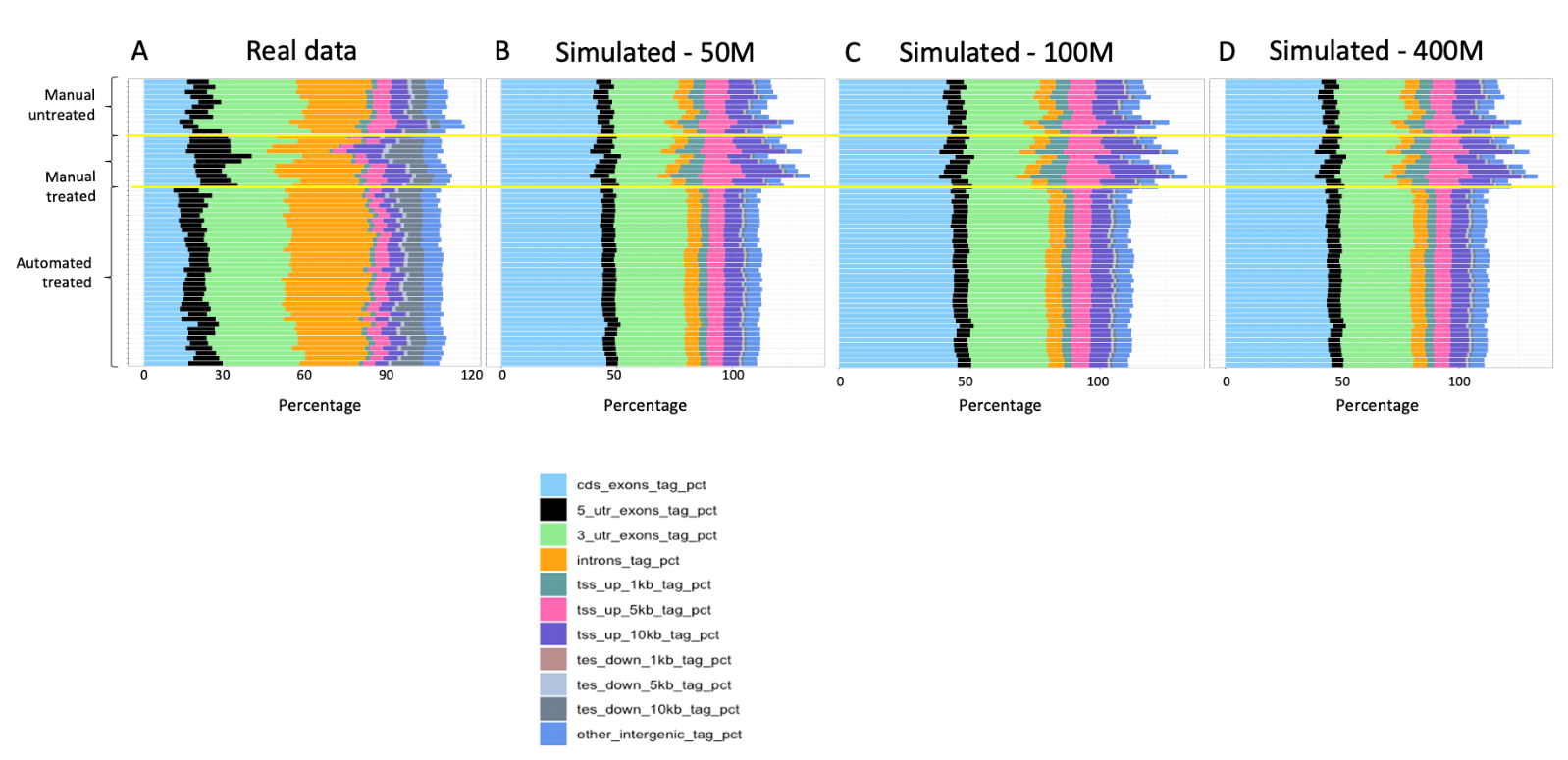


**Supplementary Figure 5.** RSeQC read distribution of the real data (A) versus the simulated data at 50M reads (B), 100M reads (C) and 400M reads (D).


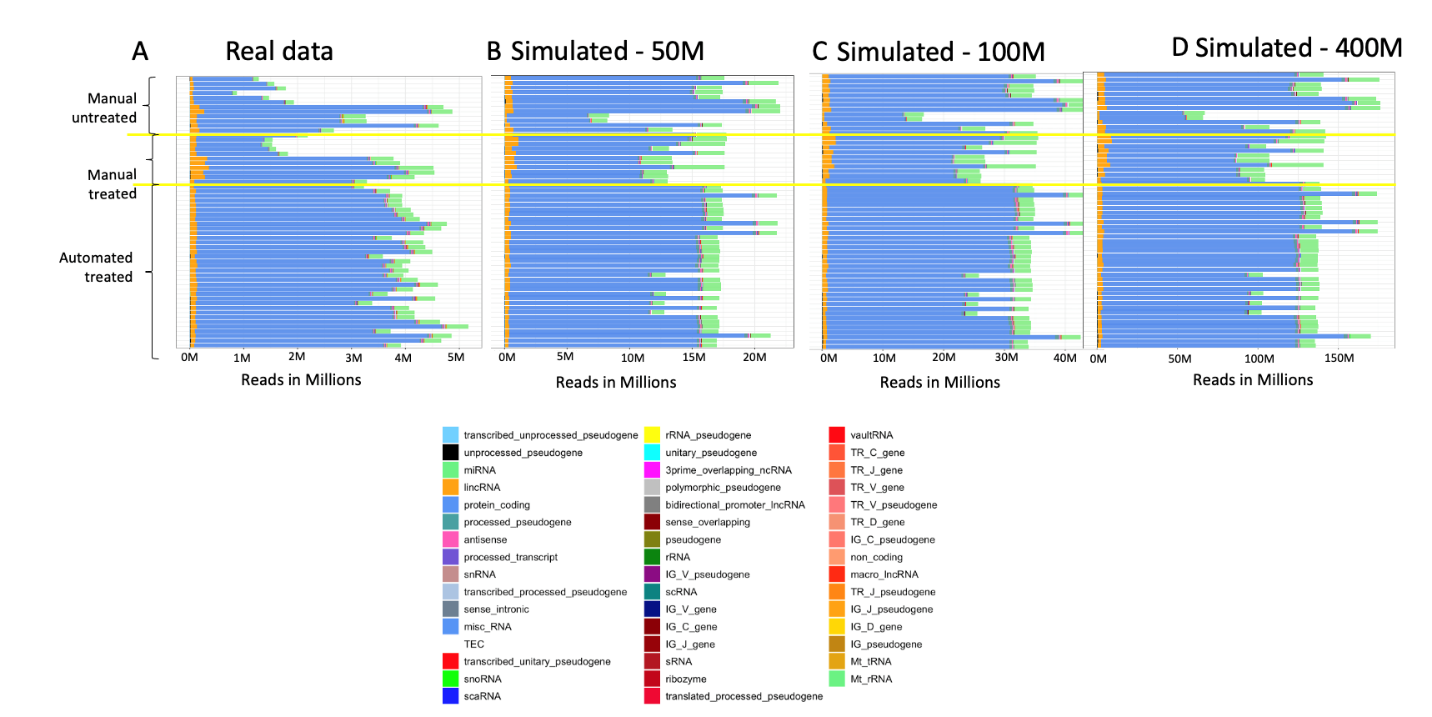


**Supplementary Figure 6.** FeatureCounts Genetypes read distribution of the real data (A) versus the simulated data at 50M reads (B), 100M reads (C) and 400M reads (D).

**Supplementary Figure 7.** Principal component analysis according to the extraction method of the complete set of real data (A); the simulated data at 50M reads (B); the simulated data at 100M reads (C) and the simulated data at 400M reads (D). Principal component analysis according to the individual subjects of the complete set of real data (E); the simulated data at 50M reads (F); the simulated data at 100M reads (G) and the simulated data at 400M reads (H).
